# Structural basis for Mep2 ammonium transceptor activation by phosphorylation

**DOI:** 10.1038/ncomms11337

**Published:** 2016-04-18

**Authors:** Bert van den Berg, Anupama Chembath, Damien Jefferies, Arnaud Basle, Syma Khalid, Julian C. Rutherford

**Affiliations:** 1 Institute for Cell and Molecular Biosciences, The Medical School, Newcastle University, Newcastle upon Tyne NE2 4HH, UK; 2 School of Chemistry, University of Southampton, Highfield Campus, Southampton SO17 1BJ, UK

## Abstract

Mep2 proteins are fungal transceptors that play an important role as ammonium sensors in fungal development. Mep2 activity is tightly regulated by phosphorylation, but how this is achieved at the molecular level is not clear. Here we report X-ray crystal structures of the Mep2 orthologues from *Saccharomyces cerevisiae* and *Candida albicans* and show that under nitrogen-sufficient conditions the transporters are not phosphorylated and present in closed, inactive conformations. Relative to the open bacterial ammonium transporters, non-phosphorylated Mep2 exhibits shifts in cytoplasmic loops and the C-terminal region (CTR) to occlude the cytoplasmic exit of the channel and to interact with His2 of the twin-His motif. The phosphorylation site in the CTR is solvent accessible and located in a negatively charged pocket ∼30 Å away from the channel exit. The crystal structure of phosphorylation-mimicking Mep2 variants from *C. albicans* show large conformational changes in a conserved and functionally important region of the CTR. The results allow us to propose a model for regulation of eukaryotic ammonium transport by phosphorylation.

## Abstract (web summary)

Mep2 proteins are tightly regulated fungal ammonium transporters. Here, the authors report the crystal structures of closed states of Mep2 proteins and propose a model for their regulation by comparing them with the open ammonium transporters of bacteria.

## Graphical abstract

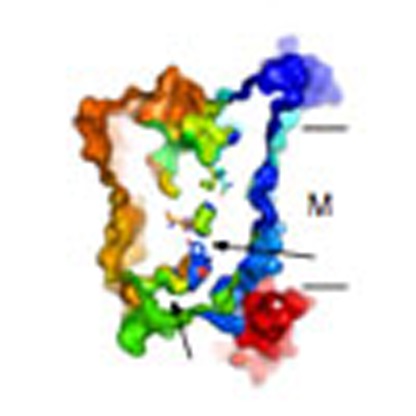

Transceptors are membrane proteins that function not only as transporters but also as receptors/sensors during nutrient sensing to activate downstream signalling pathways^1^. A common feature of transceptors is that they are induced when cells are starved for their substrate. While most studies have focused on the *Saccharomyces cerevisiae* transceptors for phosphate (Pho84), amino acids (Gap1) and ammonium (Mep2), transceptors are found in higher eukaryotes as well (for example, the mammalian SNAT2 amino-acid transporter and the GLUT2 glucose transporter)^2^. One of the most important unresolved questions in the field is how the transceptors couple to downstream signalling pathways. One hypothesis is that downstream signalling is dependent on a specific conformation of the transporter^2^.

Mep2 (methylammonium (MA) permease) proteins are ammonium transceptors that are ubiquitous in fungi. They belong to the Amt/Mep/Rh family of transporters that are present in all kingdoms of life and they take up ammonium from the extracellular environment^3^. Fungi typically have more than one Mep paralogue, for example, Mep1-3 in *S. cerevisiae*^4^. Of these, only Mep2 proteins function as ammonium receptors/sensors in fungal development^5^. Under conditions of nitrogen limitation, Mep2 initiates a signalling cascade that results in a switch from the yeast form to filamentous (pseudohyphal) growth^6^ that may be required for fungal pathogenicity^7^. As is the case for other transceptors, it is not clear how Mep2 interacts with downstream signalling partners, but the protein kinase A and mitogen-activated protein kinase pathways have been proposed as downstream effectors of Mep2 (refs 6, 8, 9). Compared with Mep1 and Mep3, Mep2 is highly expressed and functions as a low-capacity, high-affinity transporter in the uptake of MA^10^. In addition, Mep2 is also important for uptake of ammonium produced by growth on other nitrogen sources^11^.

With the exception of the human RhCG structure^12^, no structural information is available for eukaryotic ammonium transporters. By contrast, several bacterial Amt orthologues have been characterized in detail via high-resolution crystal structures and a number of molecular dynamics (MD) studies^13^,^14^,^15^,^16^,^17^,^18^,^19^. All the solved structures including that of RhCG are very similar, establishing the basic architecture of ammonium transporters. The proteins form stable trimers, with each monomer having 11 transmembrane (TM) helices and a central channel for the transport of ammonium. All structures show the transporters in open conformations. Intriguingly, fundamental questions such as the nature of the transported substrate and the transport mechanism are still controversial. Where earlier studies favoured the transport of ammonia gas^13^,^20^, recent data and theoretical considerations suggest that Amt/Mep proteins are instead active, electrogenic transporters of either NH_4_^+^ (uniport) or NH_3_/H^+^ (symport)^15^,^17^,^21^,^22^,^23^,^24^. A highly conserved pair of channel-lining histidine residues dubbed the twin-His motif may serve as a proton relay system while NH_3_ moves through the channel during NH_3_/H^+^ symport^16^.

Ammonium transport is tightly regulated. In animals, this is due to toxicity of elevated intracellular ammonium levels^25^, whereas for microorganisms ammonium is a preferred nitrogen source. In bacteria, *amt* genes are present in an operon with *glnK*, encoding a P_II_-like signal transduction class protein^26^. By binding tightly to Amt proteins without inducing a conformational change in the transporter^27^, GlnK sterically blocks ammonium conductance when nitrogen levels are sufficient. Under conditions of nitrogen limitation, GlnK becomes uridylated, blocking its ability to bind and inhibit Amt proteins^28^. Importantly, eukaryotes do not have GlnK orthologues and have a different mechanism for regulation of ammonium transport activity. In plants, transporter phosphorylation and dephosphorylation are known to regulate activity^29^. In *S. cerevisiae*, phosphorylation of Ser457 within the C-terminal region (CTR) in the cytoplasm was recently proposed to cause Mep2 opening, possibly via inducing a conformational change^30^.

To elucidate the mechanism of Mep2 transport regulation, we present here X-ray crystal structures of the Mep2 transceptors from *S. cerevisiae* and *C. albicans*. The structures are similar to each other but show considerable differences to all other ammonium transporter structures. The most striking difference is the fact that the Mep2 proteins have closed conformations. The putative phosphorylation site is solvent accessible and located in a negatively charged pocket ∼30 Å away from the channel exit. The channels of phosphorylation-mimicking mutants of *C. albicans* Mep2 are still closed but show large conformational changes within a conserved part of the CTR. Together with a structure of a C-terminal Mep2 variant lacking the segment containing the phosphorylation site, the results allow us to propose a structural model for phosphorylation-based regulation of eukaryotic ammonium transport.

## Results

### General architecture of Mep2 ammonium transceptors

**Figure 1:**
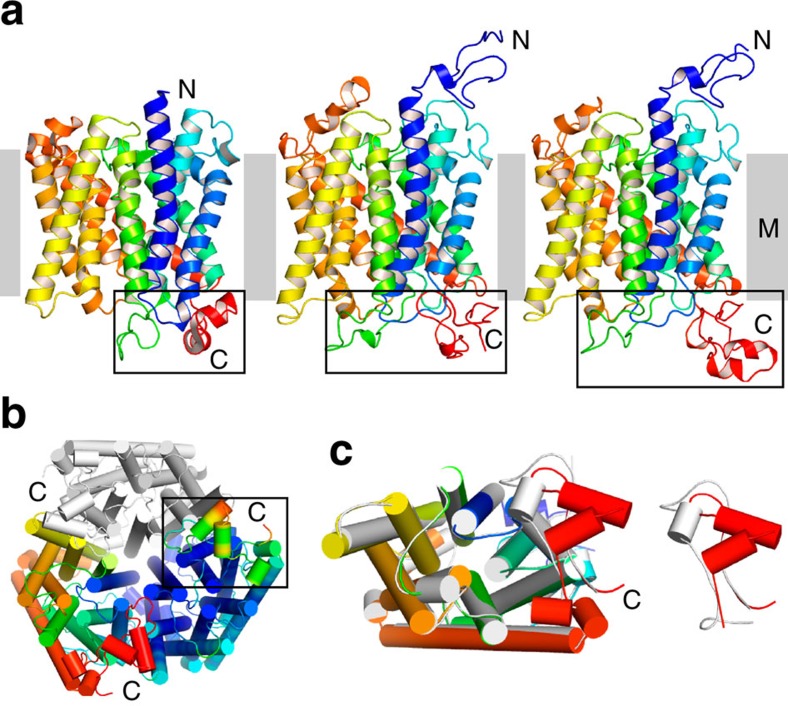
X-ray crystal structures of Mep2 transceptors. (**a**) Monomer cartoon models viewed from the side for (left) *A. fulgidus* Amt-1 (PDB ID 2B2H), *S. cerevisiae* Mep2 (middle) and *C. albicans* Mep2 (right). The cartoons are in rainbow representation. The region showing ICL1 (blue), ICL3 (green) and the CTR (red) is boxed for comparison. (**b**) CaMep2 trimer viewed from the intracellular side (right). One monomer is coloured as in **a** and one monomer is coloured by B-factor (blue, low; red; high). The CTR is boxed. (**c**) Overlay of ScMep2 (grey) and CaMep2 (rainbow), illustrating the differences in the CTRs. All structure figures were generated with Pymol^62^.

The Mep2 protein of *S. cerevisiae* (ScMep2) was overexpressed in *S. cerevisiae* in high yields, enabling structure determination by X-ray crystallography using data to 3.2 Å resolution by molecular replacement (MR) with the archaebacterial Amt-1 structure (see Methods section). Given that the modest resolution of the structure and the limited detergent stability of ScMep2 would likely complicate structure–function studies, several other fungal Mep2 orthologues were subsequently overexpressed and screened for diffraction-quality crystals. Of these, Mep2 from *C. albicans* (CaMep2) showed superior stability in relatively harsh detergents such as nonyl-glucoside, allowing structure determination in two different crystal forms to high resolution (up to 1.5 Å). Despite different crystal packing (Supplementary Table 1), the two CaMep2 structures are identical to each other and very similar to ScMep2 (C_*α*_ r.m.s.d. (root mean square deviation)=0.7 Å for 434 residues), with the main differences confined to the N terminus and the CTR (Fig. 1). Electron density is visible for the entire polypeptide chains, with the exception of the C-terminal 43 (ScMep2) and 25 residues (CaMep2), which are poorly conserved and presumably disordered. Both Mep2 proteins show the archetypal trimeric assemblies in which each monomer consists of 11 TM helices surrounding a central pore. Important functional features such as the extracellular ammonium binding site, the Phe gate and the twin-His motif within the hydrophobic channel are all very similar to those present in the bacterial transporters and RhCG^12^,^13^,^14^,^15^,^16^,^17^,^18^,^19^. In the remainder of the manuscript, we will specifically discuss CaMep2 due to the superior resolution of the structure. Unless specifically stated, the drawn conclusions also apply to ScMep2.

**Figure 2:**
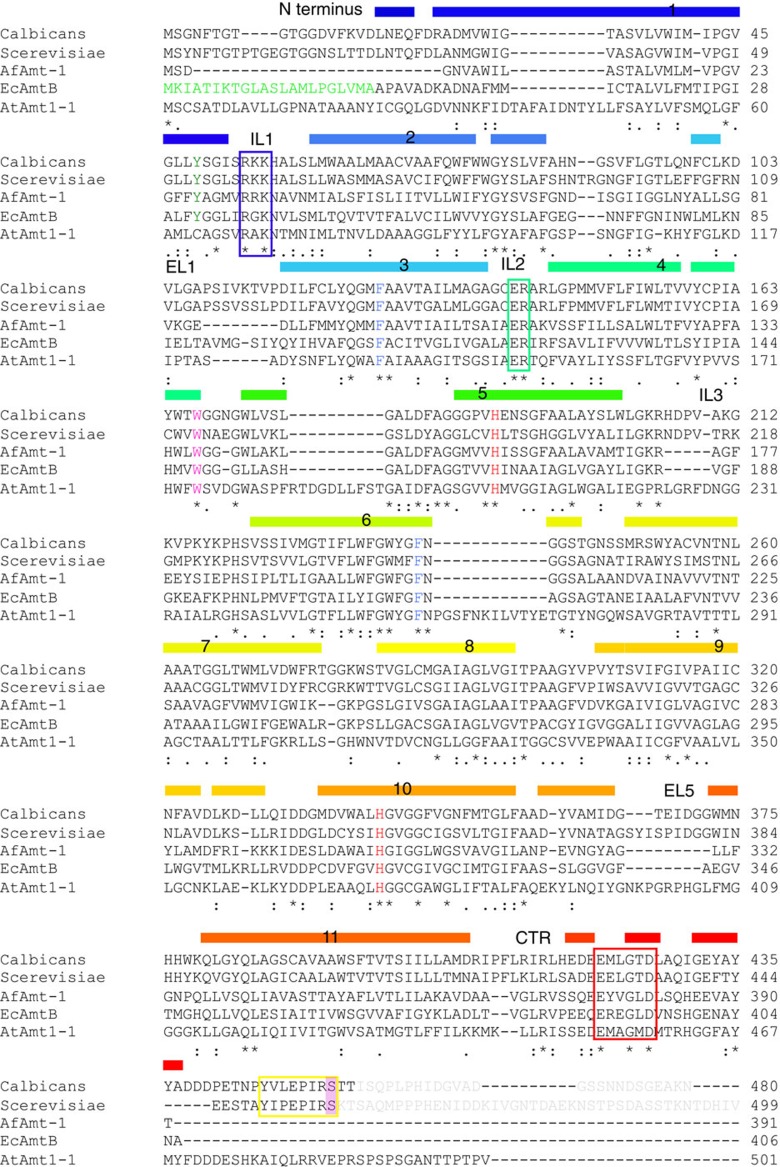
Sequence conservation in ammonium transporters. ClustalW alignment of CaMep2, ScMep2, *A. fulgidus* Amt-1, *E. coli* AmtB and *A. thaliana* Amt-1;1. The secondary structure elements observed for CaMep2 are indicated, with the numbers corresponding to the centre of the TM segment. Important regions are labelled. The conserved RxK motif in ICL1 is boxed in blue, the ER motif in ICL2 in cyan, the conserved ExxGxD motif of the CTR in red and the AI region in yellow. Coloured residues are functionally important and correspond to those of the Phe gate (blue), the binding site Trp residue (magenta) and the twin-His motif (red). The Npr1 kinase site in the AI region is highlighted pink. The grey sequences at the C termini of CaMep2 and ScMep2 are not visible in the structures and are likely disordered.

**Figure 3:**
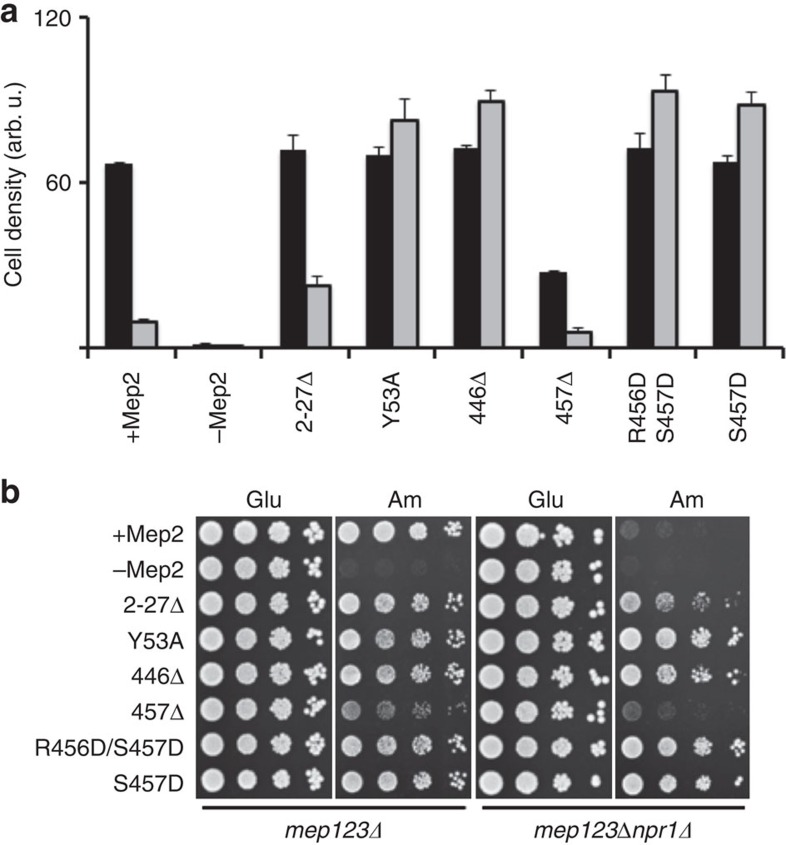
Growth of ScMep2 variants on low ammonium medium. (**a**) The triple *mepΔ* strain (black) and triple *mepΔ npr1Δ* strain (grey) containing plasmids expressing WT and variant ScMep2 were grown on minimal medium containing 1 mM ammonium sulphate. The quantified cell density reflects logarithmic growth after 24 h. Error bars are the s.d. for three replicates of each strain (**b**) The strains used in **a** were also serially diluted and spotted onto minimal agar plates containing glutamate (0.1%) or ammonium sulphate (1 mM), and grown for 3 days at 30 °C.

While the overall architecture of Mep2 is similar to that of the prokaryotic transporters (C_*α*_ r.m.s.d. with Amt-1=1.4 Å for 361 residues), there are large differences within the N terminus, intracellular loops (ICLs) ICL1 and ICL3, and the CTR. The N termini of the Mep2 proteins are ∼20–25 residues longer compared with their bacterial counterparts (Figs 1 and 2), substantially increasing the size of the extracellular domain. Moreover, the N terminus of one monomer interacts with the extended extracellular loop ECL5 of a neighbouring monomer. Together with additional, smaller differences in other extracellular loops, these changes generate a distinct vestibule leading to the ammonium binding site that is much more pronounced than in the bacterial proteins. The N-terminal vestibule and the resulting inter-monomer interactions likely increase the stability of the Mep2 trimer, in support of data for plant AMT proteins^31^. However, given that an N-terminal deletion mutant (2-27Δ) grows as well as wild-type (WT) Mep2 on minimal ammonium medium (Fig. 3 and Supplementary Fig. 1), the importance of the N terminus for Mep2 activity is not clear.

### Mep2 channels are closed by a two-tier channel block

**Figure 4:**
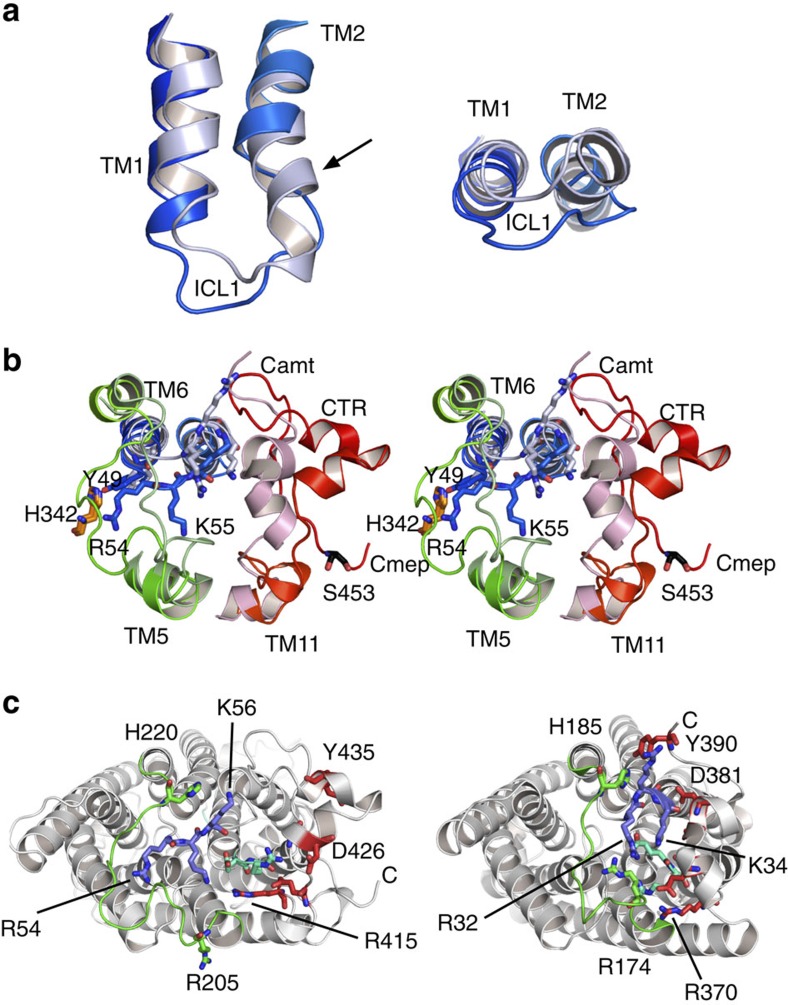
Structural differences between Mep2 and bacterial ammonium transporters. (**a**) ICL1 in AfAmt-1 (light blue) and CaMep2 (dark blue), showing unwinding and inward movement in the fungal protein. (**b**) Stereo diagram viewed from the cytosol of ICL1, ICL3 (green) and the CTR (red) in AfAmt-1 (light colours) and CaMep2 (dark colours). The side chains of residues in the RxK motif as well as those of Tyr49 and His342 are labelled. The numbering is for CaMep2. (**c**) Conserved residues in ICL1-3 and the CTR. Views from the cytosol for CaMep2 (left) and AfAmt-1, highlighting the large differences in conformation of the conserved residues in ICL1 (RxK motif; blue), ICL2 (ER motif; cyan), ICL3 (green) and the CTR (red). The labelled residues are analogous within both structures. In **b** and **c**, the centre of the trimer is at top.

**Figure 5:**
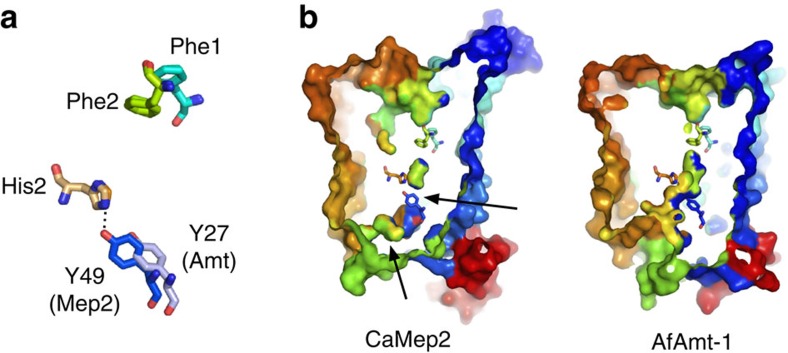
Channel closures in Mep2. (**a**) Stereo superposition of AfAmt-1 and CaMep2 showing the residues of the Phe gate, His2 of the twin-His motif and the tyrosine residue Y49 in TM1 that forms a hydrogen bond with His2 in CaMep2. (**b**) Surface views from the side in rainbow colouring, showing the two-tier channel block (indicated by the arrows) in CaMep2.

The largest differences between the Mep2 structures and the other known ammonium transporter structures are located on the intracellular side of the membrane. In the vicinity of the Mep2 channel exit, the cytoplasmic end of TM2 has unwound, generating a longer ICL1 even though there are no insertions in this region compared to the bacterial proteins (Figs 2 and 4). ICL1 has also moved inwards relative to its position in the bacterial Amts. The largest backbone movements of equivalent residues within ICL1 are ∼10 Å, markedly affecting the conserved basic RxK motif (Fig. 4). The head group of Arg54 has moved ∼11 Å relative to that in Amt-1, whereas the shift of the head group of the variable Lys55 residue is almost 20 Å. The side chain of Lys56 in the basic motif points in an opposite direction in the Mep2 structures compared with that of, for example, Amt-1 (Fig. 4). In addition to changing the RxK motif, the movement of ICL1 has another, crucial functional consequence. At the C-terminal end of TM1, the side-chain hydroxyl group of the relatively conserved Tyr49 (Tyr53 in ScMep2) makes a strong hydrogen bond with the ɛ_2_ nitrogen atom of the absolutely conserved His342 of the twin-His motif (His348 in ScMep2), closing the channel (Figs 4 and 5). In bacterial Amt proteins, this Tyr side chain is rotated ∼4 Å away as a result of the different conformation of TM1, leaving the channel open and the histidine available for its putative role in substrate transport (Supplementary Fig. 2)^16^.

Compared with ICL1, the backbone conformational changes observed for the neighbouring ICL2 are smaller, but large shifts are nevertheless observed for the conserved residues Glu140 and Arg141 (Fig. 4). Finally, the important ICL3 linking the pseudo-symmetrical halves (TM1-5 and TM6-10) of the transporter is also shifted up to ∼10 Å and forms an additional barrier that closes the channel on the cytoplasmic side (Fig. 5). This two-tier channel block likely ensures that very little ammonium transport will take place under nitrogen-sufficient conditions. The closed state of the channel might also explain why no density, which could correspond to ammonium (or water), is observed in the hydrophobic part of the Mep2 channel close to the twin-His motif. Significantly, this is also true for ScMep2, which was crystallized in the presence of 0.2 M ammonium ions (see Methods section).

The final region in Mep2 that shows large differences compared with the bacterial transporters is the CTR. In Mep2, the CTR has moved away and makes relatively few contacts with the main body of the transporter, generating a more elongated protein (Figs 1 and 4). By contrast, in the structures of bacterial proteins, the CTR is docked tightly onto the N-terminal half of the transporters (corresponding to TM1-5), resulting in a more compact structure. This is illustrated by the positions of the five universally conserved residues within the CTR, that is, Arg415(370), Glu421(376), Gly424(379), Asp426(381) and Tyr 435(390) in CaMep2(Amt-1) (Fig. 2). These residues include those of the ‘ExxGxD' motif, which when mutated generate inactive transporters^32^,^33^. In Amt-1 and other bacterial ammonium transporters, these CTR residues interact with residues within the N-terminal half of the protein. On one side, the Tyr390 hydroxyl in Amt-1 is hydrogen bonded with the side chain of the conserved His185 at the C-terminal end of loop ICL3. At the other end of ICL3, the backbone carbonyl groups of Gly172 and Lys173 are hydrogen bonded to the side chain of Arg370. Similar interactions were also modelled in the active, non-phosphorylated plant AtAmt-1;1 structure (for example, Y467-H239 and D458-K71)^33^. The result of these interactions is that the CTR ‘hugs' the N-terminal half of the transporters (Fig. 4). Also noteworthy is Asp381, the side chain of which interacts strongly with the positive dipole on the N-terminal end of TM2. This interaction in the centre of the protein may be particularly important to stabilize the open conformations of ammonium transporters. In the Mep2 structures, none of the interactions mentioned above are present.

### Phosphorylation target site is at the periphery of Mep2

Recently Boeckstaens *et al.* provided evidence that Ser457 in ScMep2 (corresponding to Ser453 in CaMep2) is phosphorylated by the TORC1 effector kinase Npr1 under nitrogen-limiting conditions^30^. In the absence of Npr1, plasmid-encoded WT Mep2 in a *S. cerevisiae mep1-3Δ* strain (triple *mepΔ*) does not allow growth on low concentrations of ammonium, suggesting that the transporter is inactive (Fig. 3 and Supplementary Fig. 1)^30^. Conversely, the phosphorylation-mimicking S457D variant is active both in the triple *mepΔ* background and in a triple *mepΔ npr1Δ* strain (Fig. 3)^30^. Mutation of other potential phosphorylation sites in the CTR did not support growth in the *npr1Δ* background^30^. Collectively, these data suggest that phosphorylation of Ser457 opens the Mep2 channel to allow ammonium uptake. Ser457 is located in a part of the CTR that is conserved in a subgroup of Mep2 proteins, but which is not present in bacterial proteins (Fig. 2). This segment (residues 450–457 in ScMep2 and 446–453 in CaMep2) was dubbed an autoinhibitory (AI) region based on the fact that its removal generates an active transporter in the absence of Npr1 (Fig. 3)^30^.

**Figure 6:**
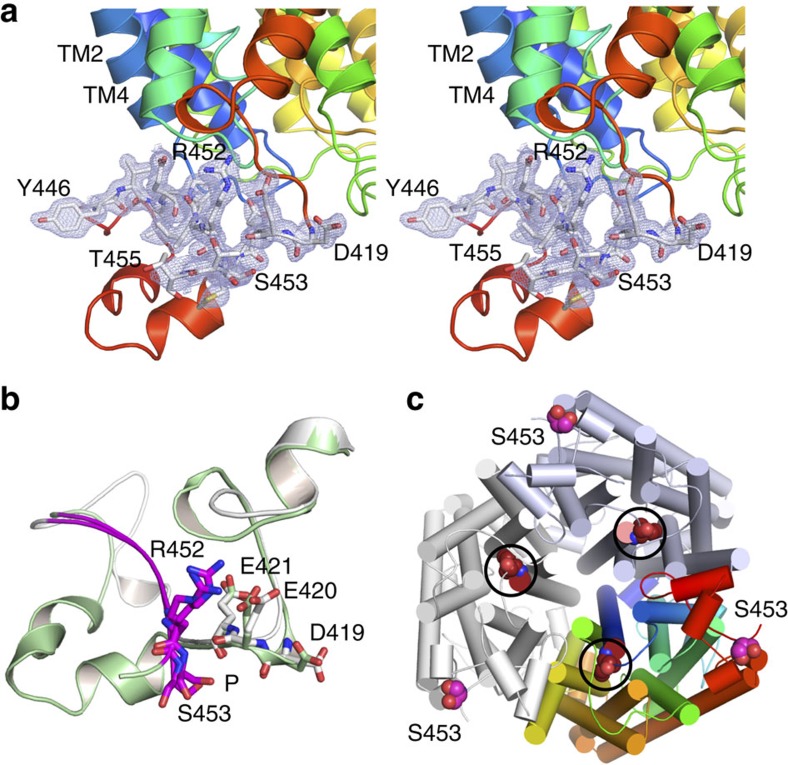
The Npr1 kinase target Ser453 is dephosphorylated and located in an electronegative pocket. (**a**) Stereoviews of CaMep2 showing 2F_o_–F_c_ electron density (contoured at 1.0 σ) for CTR residues Asp419-Met422 and for Tyr446-Thr455 of the AI region. For clarity, the residues shown are coloured white, with oxygen atoms in red and nitrogen atoms in blue. The phosphorylation target residue Ser453 is labelled in bold. (**b**) Overlay of the CTRs of ScMep2 (grey) and CaMep2 (green), showing the similar electronegative environment surrounding the phosphorylation site (P). The AI regions are coloured magenta. (**c**) Cytoplasmic view of the Mep2 trimer indicating the large distance between Ser453 and the channel exits (circles; Ile52 lining the channel exit is shown).

Where is the AI region and the Npr1 phosphorylation site located? Our structures reveal that surprisingly, the AI region is folded back onto the CTR and is not located near the centre of the trimer as expected from the bacterial structures (Fig. 4). The AI region packs against the cytoplasmic ends of TM2 and TM4, physically linking the main body of the transporter with the CTR via main chain interactions and side-chain interactions of Val447, Asp449, Pro450 and Arg452 (Fig. 6). The AI regions have very similar conformations in CaMep2 and ScMep2, despite considerable differences in the rest of the CTR (Fig. 6). Strikingly, the Npr1 target serine residue is located at the periphery of the trimer, far away (∼30 Å) from any channel exit (Fig. 6). Despite its location at the periphery of the trimer, the electron density for the serine is well defined in both Mep2 structures and corresponds to the non-phosphorylated state (Fig. 6). This makes sense since the proteins were expressed in rich medium and confirms the recent suggestion by Boeckstaens *et al.* that the non-phosphorylated form of Mep2 corresponds to the inactive state^30^. For ScMep2, Ser457 is the most C-terminal residue for which electron density is visible, indicating that the region beyond Ser457 is disordered. In CaMep2, the visible part of the sequence extends for two residues beyond Ser453 (Fig. 6). The peripheral location and disorder of the CTR beyond the kinase target site should facilitate the phosphorylation by Npr1. The disordered part of the CTR is not conserved in ammonium transporters (Fig. 2), suggesting that it is not important for transport. Interestingly, a ScMep2 457Δ truncation mutant in which a His-tag directly follows Ser457 is highly expressed but has low activity (Fig. 3 and Supplementary Fig. 1b), suggesting that the His-tag interferes with phosphorylation by Npr1. The same mutant lacking the His-tag has WT properties (Supplementary Fig. 1b), confirming that the region following the phosphorylation site is dispensable for function.

### Mep2 lacking the AI region is conformationally heterogeneous

**Figure 7:**
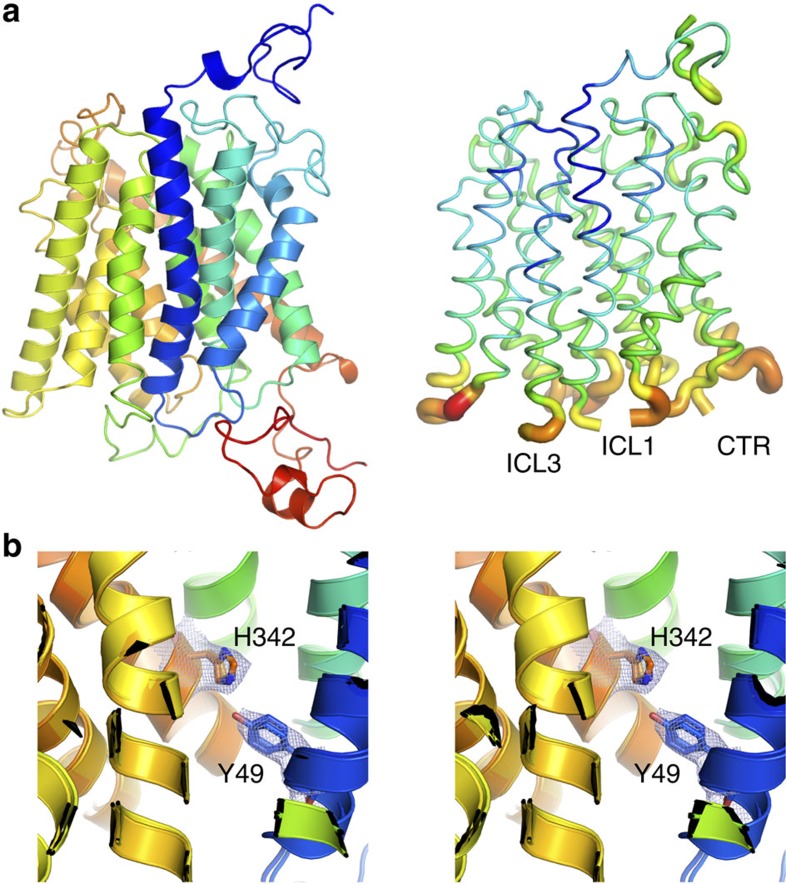
Effect of removal of the AI region on Mep2 structure. (**a**) Side views for WT CaMep2 (left) and the truncation mutant 442Δ (right). The latter is shown as a putty model according to B-factors to illustrate the disorder in the protein on the cytoplasmic side. Missing regions are labelled. (**b**) Stereo superpositions of WT CaMep2 and the truncation mutant. 2F_o_–F_c_ electron density (contoured at 1.0 σ) for residues Tyr49 and His342 is shown for the truncation mutant.

Given that Ser457/453 is far from any channel exit (Fig. 6), the crucial question is how phosphorylation opens the Mep2 channel to generate an active transporter. Boeckstaens *et al.* proposed that phosphorylation does not affect channel activity directly, but instead relieves inhibition by the AI region. The data behind this hypothesis is the observation that a ScMep2 449-485Δ deletion mutant lacking the AI region is highly active in MA uptake both in the triple *mepΔ* and triple *mepΔ npr1Δ* backgrounds, implying that this Mep2 variant has a constitutively open channel^30^. We obtained a similar result for ammonium uptake by the 446Δ mutant (Fig. 3), supporting the data from Marini *et al.* We then constructed and purified the analogous CaMep2 442Δ truncation mutant and determined the crystal structure using data to 3.4 Å resolution. The structure shows that removal of the AI region markedly increases the dynamics of the cytoplasmic parts of the transporter. This is not unexpected given the fact that the AI region bridges the CTR and the main body of Mep2 (Fig. 6). Density for ICL3 and the CTR beyond residue Arg415 is missing in the 442Δ mutant, and the density for the other ICLs including ICL1 is generally poor with visible parts of the structure having high B-factors (Fig. 7). Interestingly, however, the Tyr49-His342 hydrogen bond that closes the channel in the WT protein is still present (Fig. 7 and Supplementary Fig. 2). Why then does this mutant appear to be constitutively active? We propose two possibilities. The first one is that the open state is disfavoured by crystallization because of lower stability or due to crystal packing constraints. The second possibility is that the Tyr–His hydrogen bond has to be disrupted by the incoming substrate to open the channel. The latter model would fit well with the NH_3_/H^+^ symport model in which the proton is relayed by the twin-His motif^14^. The importance of the Tyr–His hydrogen bond is underscored by the fact that its removal in the ScMep2 Y53A mutant results in a constitutively active transporter (Fig. 3).

### Phosphorylation causes a conformational change in the CTR

**Figure 8:**
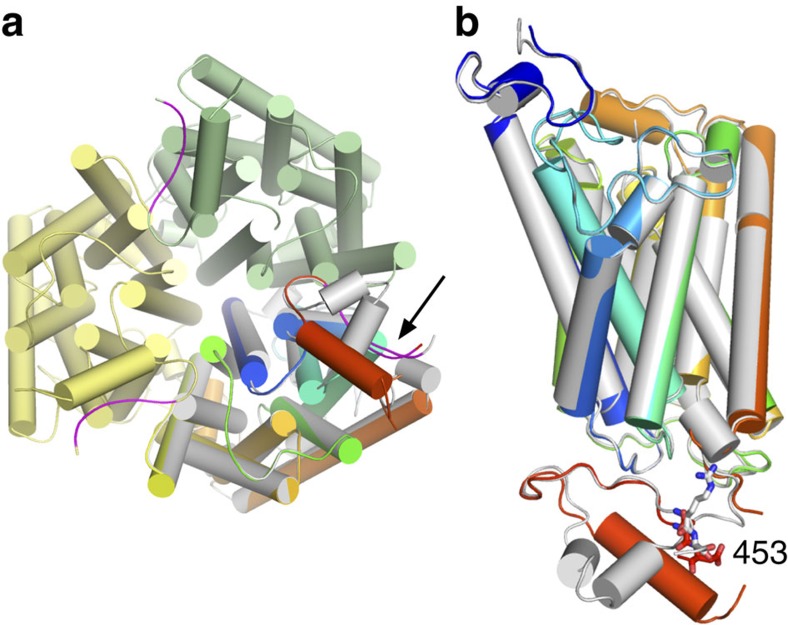
Phosphorylation causes conformational changes in the CTR. (**a**) Cytoplasmic view of the DD mutant trimer, with WT CaMep2 superposed in grey for one of the monomers. The arrow indicates the phosphorylation site. The AI region is coloured magenta. (**b**) Monomer side-view superposition of WT CaMep2 and the DD mutant, showing the conformational change and disorder around the ExxGxD motif. Side chains for residues 452 and 453 are shown as stick models.

Do the Mep2 structures provide any clues regarding the potential effect of phosphorylation? The side-chain hydroxyl of Ser457/453 is located in a well-defined electronegative pocket that is solvent accessible (Fig. 6). The closest atoms to the serine hydroxyl group are the backbone carbonyl atoms of Asp419, Glu420 and Glu421, which are 3–4 Å away. We therefore predict that phosphorylation of Ser453 will result in steric clashes as well as electrostatic repulsion, which in turn might cause substantial conformational changes within the CTR. To test this hypothesis, we determined the structure of the phosphorylation-mimicking R452D/S453D protein (hereafter termed ‘DD mutant'), using data to a resolution of 2.4 Å. The additional mutation of the arginine preceding the phosphorylation site was introduced (i) to increase the negative charge density and make it more comparable to a phosphate at neutral pH, and (ii) to further destabilize the interactions of the AI region with the main body of the transporter (Fig. 6). The ammonium uptake activity of the *S. cerevisiae* version of the DD mutant is the same as that of WT Mep2 and the S453D mutant, indicating that the mutations do not affect transporter functionality in the triple *mep*Δ background (Fig. 3). Unexpectedly, the AI segment containing the mutated residues has only undergone a slight shift compared with the WT protein (Fig. 8 and Supplementary Fig. 3). By contrast, the conserved part of the CTR has undergone a large conformational change involving formation of a 12-residue-long α-helix from Leu427 to Asp438. In addition, residues Glu420-Leu423 including Glu421 of the ExxGxD motif are now disordered (Fig. 8 and Supplementary Fig. 3). Overall, ∼20 residues are affected by the introduced mutations. This is the first time a large conformational change has been observed in an ammonium transporter as a result of a mutation, and confirms previous hypotheses that phosphorylation causes structural changes in the CTR^29^,^30^,^33^. To exclude the possibility that the additional R452D mutation is responsible for the observed changes, we also determined the structure of the ‘single D' S453D mutant. As shown in Supplementary Fig. 4, the consequence of the single D mutation is very similar to that of the DD substitution, with conformational changes and increased dynamics confined to the conserved part of the CTR (Supplementary Fig. 4). To supplement the crystal structures, we also performed modelling and MD studies of WT CaMep2, the DD mutant and phosphorylated protein (S453J). In the WT structure, the acidic residues Asp419, Glu420 and Glu421 are within hydrogen bonding distance of Ser453. After 200 ns of MD simulation, the interactions between these residues and Ser453 remain intact. The protein backbone has an average r.m.s.d. of only ∼3 Å during the 200-ns simulation, indicating that the protein is stable. There is flexibility in the side chains of the acidic residues so that they are able to form stable hydrogen bonds with Ser453. In particular, persistent hydrogen bonds are observed between the Ser453 hydroxyl group and the acidic group of Glu420, and also between the amine group of Ser453 and the backbone carbonyl of Glu420 (Supplementary Fig. 5). The DD mutant is also stable during the simulations, but the average backbone r.m.s.d of ∼3.6 Å suggests slightly more conformational flexibility than WT. As the simulation proceeds, the side chains of the acidic residues move away from Asp452 and Asp453, presumably to avoid electrostatic repulsion. For example, the distance between the Asp453 acidic oxygens and the Glu420 acidic oxygens increases from ∼7 to >22 Å after 200 ns simulations, and thus these residues are not interacting. The protein is structurally stable throughout the simulation with little deviation in the other parts of the protein. Finally, the S453J mutant is also stable throughout the 200-ns simulation and has an average backbone deviation of ∼3.8 Å, which is similar to the DD mutant. The movement of the acidic residues away from Arg452 and Sep453 is more pronounced in this simulation in comparison with the movement away from Asp452 and Asp453 in the DD mutant. The distance between the phosphate of Sep453 and the acidic oxygen atoms of Glu420 is initially ∼11 Å, but increases to >30 Å after 200 ns. The short helix formed by residues Leu427 to Asp438 unravels during the simulations to a disordered state. The remainder of the protein is not affected (Supplementary Fig. 5). Thus, the MD simulations support the notion from the crystal structures that phosphorylation generates conformational changes in the conserved part of the CTR. However, the conformational changes for the phosphomimetic mutants in the crystals are confined to the CTR (Fig. 8), and the channels are still closed (Supplementary Fig. 2). One possible explanation is that the mutants do not accurately mimic a phosphoserine, but the observation that the S453D and DD mutants are fully active in the absence of Npr1 suggests that the mutations do mimic the effect of phosphorylation (Fig. 3). The fact that the S453D structure was obtained in the presence of 10 mM ammonium ions suggests that the crystallization process favours closed states of the Mep2 channels.

## Discussion

Knowledge about ammonium transporter structure has been obtained from experimental and theoretical studies on bacterial family members. In addition, a number of biochemical and genetic studies are available for bacterial, fungal and plant proteins. These efforts have advanced our knowledge considerably but have not yet yielded atomic-level answers to several important mechanistic questions, including how ammonium transport is regulated in eukaryotes and the mechanism of ammonium signalling. In *Arabidopsis* *thaliana* Amt-1;1, phosphorylation of the CTR residue T460 under conditions of high ammonium inhibits transport activity, that is, the default (non-phosphorylated) state of the plant transporter is open. Interestingly, phosphomimetic mutations introduced into one monomer inactivate the entire trimer, indicating that (i) heterotrimerization occurs and (ii) the CTR mediates allosteric regulation of ammonium transport activity via phosphorylation^33^,^34^. Owing to the lack of structural information for plant AMTs, the details of channel closure and inter-monomer crosstalk are not yet clear. Contrasting with the plant transporters, the inactive states of Mep2 proteins under conditions of high ammonium are non-phosphorylated, with channels that are closed on the cytoplasmic side. The reason why similar transporters such as *A. thaliana* Amt-1;1 and Mep2 are regulated in opposite ways by phosphorylation (inactivation in plants and activation in fungi) is not known. In fungi, preventing ammonium entry via channel closure in ammonium transporters would be one way to alleviate ammonium toxicity, in addition to ammonium excretion via Ato transporters and amino-acid secretion^35^.

**Figure 9:**
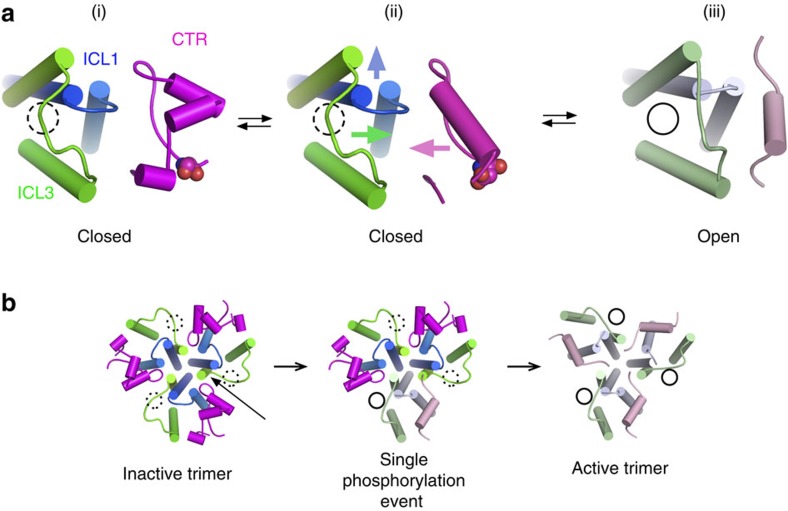
Schematic model for phosphorylation-based regulation of Mep2 ammonium transporters. (**a**) In the closed, non-phosphorylated state (i), the CTR (magenta) and ICL3 (green) are far apart with the latter blocking the intracellular channel exit (indicated with a hatched circle). Upon phosphorylation and mimicked by the CaMep2 S453D and DD mutants (ii), the region around the ExxGxD motif undergoes a conformational change that results in the CTR interacting with the inward-moving ICL3, opening the channel (full circle) (iii). The arrows depict the movements of important structural elements. The open-channel Mep2 structure is represented by archaebacterial Amt-1 and shown in lighter colours consistent with Fig. 4. As discussed in the text, similar structural arrangements may occur in plant AMTs. In this case however, the open channel corresponds to the non-phosphorylated state; phosphorylation breaks the CTR–ICL3 interactions leading to channel closure. (**b**) Model based on AMT transporter analogy^29^,^33^ showing how phosphorylation of a Mep2 monomer might allosterically open channels in the entire trimer via disruption of the interactions between the CTR and ICL3 of a neighbouring monomer (arrow).

By determining the first structures of closed ammonium transporters and comparing those structures with the permanently open bacterial proteins, we demonstrate that Mep2 channel closure is likely due to movements of the CTR and ICL1 and ICL3. More specifically, the close interactions between the CTR and ICL1/ICL3 present in open transporters are disrupted, causing ICL3 to move outwards and block the channel (Figs 4 and 9a). In addition, ICL1 has shifted inwards to contribute to the channel closure by engaging His2 from the twin-His motif via hydrogen bonding with a highly conserved tyrosine hydroxyl group. Upon phosphorylation by the Npr1 kinase in response to nitrogen limitation, the region around the conserved ExxGxD motif undergoes a conformational change that opens the channel (Fig. 9). Importantly, the structural similarities in the TM parts of Mep2 and AfAmt-1 (Fig. 5a) suggest that channel opening/closure does not require substantial changes in the residues lining the channel. How exactly the channel opens and whether opening is intra-monomeric are still open questions; it is possible that the change in the CTR may disrupt its interactions with ICL3 of the neighbouring monomer (Fig. 9b), which could result in opening of the neighbouring channel via inward movement of its ICL3. Owing to the crosstalk between monomers^33^,^34^, a single phosphorylation event might lead to opening of the entire trimer, although this has not yet been tested (Fig. 9b). Whether or not Mep2 channel opening requires, in addition to phosphorylation, disruption of the Tyr–His2 interaction by the ammonium substrate is not yet clear.

Is our model for opening and closing of Mep2 channels valid for other eukaryotic ammonium transporters? Our structural data support previous studies^30^,^31^,^32^,^33^,^34^ and clarify the central role of the CTR and cytoplasmic loops in the transition between closed and open states. However, even the otherwise highly similar Mep2 proteins of *S. cerevisiae* and *C. albicans* have different structures for their CTRs (Fig. 1 and Supplementary Fig. 6). In addition, the AI region of the CTR containing the Npr1 kinase site is conserved in only a subset of fungal transporters, suggesting that the details of the structural changes underpinning regulation vary. Nevertheless, given the central role of absolutely conserved residues within the ICL1-ICL3-CTR interaction network (Fig. 4), we propose that the structural basics of fungal ammonium transporter activation are conserved. The fact that Mep2 orthologues of distantly related fungi are fully functional in ammonium transport and signalling in *S. cerevisiae* supports this notion^36^,^37^,^38^. It should also be noted that the tyrosine residue interacting with His2 is highly conserved in fungal Mep2 orthologues, suggesting that the Tyr–His2 hydrogen bond might be a general way to close Mep2 proteins.

With regards to plant AMTs, it has been proposed that phosphorylation at T460 generates conformational changes that would close the neighbouring pore via the C terminus. This assumption was based partly on a homology model for Amt-1;1 based on the (open) archaebacterial AfAmt-1 structure, which suggested that the C terminus of Amt-1;1 would extend further to the neighbouring monomer^33^. Our Mep2 structures show that this assumption may not be correct (Fig. 4 and Supplementary Fig. 6). In addition, the considerable differences between structurally resolved CTR domains means that the exact environment of T460 in Amt-1;1 is also not known (Supplementary Fig. 6). Based on the available structural information, we consider it more likely that phosphorylation-mediated pore closure in Amt-1;1 is intra-monomeric, via disruption of the interactions between the CTR and ICL1/ICL3 (for example, Y467-H239 and D458-K71)^33^. There is generally no equivalent for CaMep2 Tyr49 in plant AMTs, indicating that a Tyr–His2 hydrogen bond as observed in Mep2 may not contribute to the closed state in plant transporters.

We propose that intra-monomeric CTR-ICL1/ICL3 interactions lie at the basis of regulation of both fungal and plant ammonium transporters; close interactions generate open channels, whereas the lack of ‘intra-' interactions leads to inactive states. The need to regulate in opposite ways may be the reason why the phosphorylation sites are in different parts of the CTR, that is, centrally located close to the ExxGxD motif in AMTs and peripherally in Mep2. In this way, phosphorylation can either lead to channel closing (in the case of AMTs) or channel opening in the case of Mep2. Our model also provides an explanation for the observation that certain mutations within the CTR completely abolish transport activity. An example of an inactivating residue is the glycine of the ExxGxD motif of the CTR. Mutation of this residue (G393 in EcAmtB; G456 in AtAmt-1;1) inactivates transporters as diverse as *Escherichia coli* AmtB and *A. thaliana* Amt-1;1 (refs 32, 33). Such mutations likely cause structural changes in the CTR that prevent close contacts between the CTR and ICL1/ICL3, thereby stabilizing a closed state that may be similar to that observed in Mep2.

Regulation and modulation of membrane transport by phosphorylation is known to occur in, for example, aquaporins^39^ and urea transporters^40^, and is likely to be a common theme for eukaryotic channels and transporters. Recently, phosphorylation was also shown to modulate substrate affinity in nitrate transporters^41^. With respect to ammonium transport, phosphorylation has thus far only been shown for *A. thaliana* AMTs and for *S. cerevisiae* Mep2 (refs 29, 30, 32, 33). However, the absence of GlnK proteins in eukaryotes suggests that phosphorylation-based regulation of ammonium transport may be widespread. Nevertheless, as discussed above, considerable differences may exist between different species.

With respect to Mep2-mediated signalling to induce pseudohyphal growth, two models have been put forward as to how this occurs and why it is specific to Mep2 proteins. In one model, signalling is proposed to depend on the nature of the transported substrate, which might be different in certain subfamilies of ammonium transporters (for example, Mep1/Mep3 versus Mep2). For example, NH_3_ uniport or symport of NH_3_/H^+^ might result in changes in local pH, but NH_4_^+^ uniport might not^10^,^21^, and this difference might determine signalling. In the other model, signalling is thought to require a distinct conformation of the Mep2 transporter occurring during the transport cycle^8^. While the current study does not specifically address the mechanism of signalling underlying pseudohyphal growth, our structures do show that Mep2 proteins can assume different conformations.

It is clear that ammonium transport across biomembranes remains a fascinating and challenging field in large part due to the unique properties of the substrate. Our Mep2 structural work now provides a foundation for future studies to uncover the details of the structural changes that occur during eukaryotic ammonium transport and signaling, and to assess the possibility to utilize small molecules to shut down ammonium sensing and downstream signalling pathways in pathogenic fungi.

## Methods

### Mep2 overexpression and purification

Ammonium transporter genes were amplified from genomic DNA or cDNA by PCR (Phusion, New England Biolabs). In both *ScMEP2* and *CaMEP2*, Asn4 was replaced by a glutamine to prevent glycosylation. In order to allow transformation of yeast by recombination, the following primer extensions were used: forward 5′-GAAAAAACCCCGGATTCTAGAACTAGTGGATCCTCC-3′ and reverse 5′-TGACTCGAGTTATGCACCGTGGTGGTGATGGTGATG-3′. These primers result in a construct that lacks the cleavable N- and C-terminal tags present in the original vector^42^, and replaces these with a C-terminal hexa-histidine tag. Recombination in yeast strain W303 *pep4Δ* was carried out using ∼50–100 ng of SmaI-digested vector 83νΔ (ref. 42) and at least a fourfold molar excess of PCR product via the lithium acetate method. Transformants were selected on SCD -His plates incubated at 30 °C. Construction of mutant *CaMEP2* genes was done using the Q5 site-directed mutagenesis kit (NEB) per manufacturer's instructions. Three CaMep2 mutants were made for crystallization: the first mutant is a C-terminal truncation mutant 442Δ, lacking residues 443–480 including the AI domain. The second mutant, R452D/S453D, mimics the protein phosphorylated at Ser453. Given that phosphate is predominantly charged −2 at physiological pH, we introduced the second aspartate residue for Arg452. However, we also constructed the ‘single D', S453D CaMep2 variant.

For expression, cells were grown in shaker flasks at 30 °C for ∼24 h in synthetic minimal medium lacking histidine and with 1% (w/v) glucose to a typical OD_600_ of 6–8. Cells were subsequently spun down for 15 min at 4,000*g* and resuspended in YP medium containing 1.5% (w/v) galactose, followed by another 16–20 h growth at 30 °C/160 r.p.m. and harvesting by centrifugation. Final OD_600_ values typically reached 18–20. Cells were lysed by bead beating (Biospec) for 5 × 1 min with 1 min intervals on ice, or by 1–2 passes through a cell disrupter operated at 35,000 p.s.i. (TS-Series 0.75 kW; Constant Systems). Membranes were collected from the suspension by centrifugation at 200,000*g* for 90 min (45Ti rotor; Beckmann Coulter). Membrane protein extraction was performed by homogenization in a 1:1 (w/w) mixture of dodecyl-β-D-maltoside and decyl-β-D-maltoside (DDM/DM) followed by stirring at 4 °C overnight. Typically, 1 g (1% w/v) of total detergent was used for membranes from 2 l of cells. The membrane extract was centrifuged for 35 min at 200,000*g* and the supernatant was loaded onto a 10-ml Nickel column (Chelating Sepharose; GE Healthcare) equilibrated in 20 mM Tris/300 mM NaCl/0.2% DDM, pH 8. The column was washed with 15 column volumes buffer containing 30 mM imidazole and eluted in 3 column volumes with 250 mM imidazole. Proteins were purified to homogeneity by gel filtration chromatography in 10 mM HEPES/100 mM NaCl/0.05% DDM, pH 7–7.5. For polishing and detergent exchange, a second gel filtration column was performed using various detergents. In the case of ScMep2, diffracting crystals were obtained only with 0.05% decyl-maltose neopentyl glycol. For the more stable CaMep2 protein, we obtained crystals in, for example, nonyl-glucoside, decyl-maltoside and octyl-glucose neopentyl glycol. Proteins were concentrated to 7–15 mg ml^−1^ using 100 kDa cutoff centrifugal devices (Millipore), flash-frozen and stored at −80 °C before use.

### Crystallization and structure determination

Crystallization screening trials by sitting drop vapour diffusion were set up at 4 and 20 °C using in-house screens and the MemGold 1 and 2 screens (Molecular Dimensions) with a Mosquito crystallization robot. Crystals were harvested directly from the initial trials or optimized by sitting or hanging drop vapour diffusion using larger drops (typically 2–3 μl total volume). Bar-shaped crystals for ScMep2 diffracting to 3.2 Å resolution were obtained from 50 mM 2-(N-morpholino)ethanesulfonic acid (MES)/0.2 M di-ammonium hydrogen phosphate/30% PEG 400, pH 6. They belong to space group P2_1_2_1_2_1_ and have nine molecules (three trimers) in the asymmetric unit (AU). Well-diffracting crystals for CaMep2 were obtained in space group P3 from 0.1 M MES/0.2 M lithium sulphate/20% PEG400, pH 6 (two molecules per AU). An additional crystal form in space group R3 was grown in 0.04 M Tris/0.04 M NaCl/27% PEG350 MME, pH 8 (one molecule per AU). Diffracting crystals for the phosporylation-mimicking CaMep2 DD mutant were obtained in space group P6_3_22 from 0.1 M sodium acetate/15–20% PEG400, pH 5 (using decyl-maltoside as detergent; one molecule per AU), while S453D mutant crystals grew in 24% PEG400/0.05 M sodium acetate, pH 5.4/0.05 M magnesium acetate tetrahydrate/10 mM NH_4_Cl (space group R3_2_; one molecule per AU). Finally, the 442Δ truncation mutant gave crystals under many different conditions, but most of these diffracted poorly or not at all. A reasonable low-resolution data set (3.4 Å resolution) was eventually obtained from a crystal grown in 24% PEG400/0.05 M sodium acetate/0.05 M magnesium acetate, pH 6.1 (space group R3_2_). Diffraction data were collected at the Diamond Light Source and processed with XDS^43^ or HKL2000 (ref. 44).

For MR, a search model was constructed with Sculptor within Phenix^45^, using a sequence alignment of ScMep2 with *Archaeoglobus fulgidus* Amt-1 (PDB ID 2B2H; ∼40% sequence identity to ScMep2). A clear solution with nine molecules (three trimers) in the AU was obtained using Phaser^46^. The model was subsequently completed by iterative rounds of manual building within Coot^47^ followed by refinement within Phenix. The structures for WT CaMep2 were solved using the best-defined monomer of ScMep2 (60% sequence identity with CaMep2) in MR with Phaser, followed by automated model building within Phenix. Finally, the structures of the three mutant CaMep2 proteins were solved using WT CaMep2 as the search model. The data collection and refinement statistics for all six solved structures have been summarized in Supplementary Tables 1 and 2.

### Growth assays

The *S. cerevisiae* haploid triple mepΔ strain (Σ1278b MATα mep1::LEU2 mep2::LEU2 mep3::G418 ura3-52) and triple mepΔ npr1Δ strain (Σ1278b MATα mep1::LEU2 mep2::LEU2 mep3::G418 npr1::NAT1 ura3-52) were generated by integrating the NAT1 resistance gene at one NPR1 locus in the diploid strain MLY131 (ref. 6), followed by isolation of individual haploid strains. Cells were grown in synthetic minimal medium with glucose (2%) as the carbon source and ammonium sulphate (1 mM) or glutamate (0.1%) as the nitrogen source. Yeast cells were transformed as described^48^. All DNA sequences encoding epitope-tagged ScMep2 and its mutant derivatives were generated by PCR and homologous recombination using the vector pRS316 (ref. 49). In each case, the ScMEP2 sequences included the ScMEP2 promoter (1 kb), the ScMEP2 terminator and sequences coding for a His-6 epitope at the C-terminal end of the protein. All Mep2-His fusions contain the N4Q mutation to prevent glycosylation of Mep2 (ref. 50). All newly generated plasmid inserts were verified by DNA sequencing. For growth assays, *S. cerevisiae* cells containing plasmids expressing ScMep2 or mutant derivatives were grown overnight in synthetic minimal glutamate medium, washed, spotted by robot onto solid agar plates and culture growth followed by time course photography. Images were then processed to quantify the growth of each strain over 3 days as described^51^.

### Protein modelling

The MODELLER (version 9.15) software package^52^ was used to build protein structures for MD simulations. This method was required to construct two complete protein models, the double mutant R452D/S453D (with the four missing residues from the X-ray structure added) and also the construct in which the mutation at position 452 is reverted to R, and D453 is replaced with a phosphoserine. The quality of these models was assessed using normalized Discrete Optimized Protein Energy (DOPE) values and the molpdf assessment function within the MODELLER package. The model R452D/S453D mutant has a molpdf assessment score of 1854.05, and a DOPE assessment score of -60920.55. The model of the S453J mutant has a molpdf assessment score of 1857.01 and a DOPE assessment score of −61032.15.

### MD simulations

WT and model structures were embedded into a pre-equilibrated lipid bilayer composed of 512 dipalmitoylphosphatidylcholine lipids using the InflateGRO2 computer programme^53^. The bilayers were then solvated with the SPC water model^54^ and counterions were added to achieve a charge neutral state. All simulations were performed with the GROMACS package (version 4.5.5)^55^, and the GROMOS96 43a1p force field. During simulation time, the temperature was maintained at 310 K using the Nosé-Hoover thermostat^56^,^57^ with a coupling constant of 0.5 ps. Pressure was maintained at 1 bar using semi-isotropic coupling with the Parrinello-Rahman barostat^58^ and a time constant of 5 ps. Electrostatic interactions were treated using the smooth particle mesh Ewald algorithm^59^ with a short-range cutoff of 0.9 nm. Van der Waals interactions were truncated at 1.4 nm with a long-range dispersion correction applied to energy and pressure. The neighbour list was updated every five steps. All bonds were constrained with the LINCS algorithm^60^, so that a 2-fs time step could be applied throughout. The phospholipid parameters for the dipalmitoylphosphatidylcholine lipids were based on the work of Berger^61^. The embedded proteins were simulated for 200 ns each; a repeat simulation was performed for each system with different initial velocities to ensure reproducibility. To keep the c.p.u. times within reasonable limits, all simulations were performed on Mep2 monomers. This is also consistent with previous simulations for *E. coli* AmtB^16^,^17^,^18^.

## Additional information

**Accession codes:** The atomic coordinates and the associated structure factors have been deposited in the Protein Data Bank (http:// www.pdbe.org) with accession codes 5AEX (ScMep2), 5AEZ(CaMep2; R3), 5AF1(CaMep2; P3), 5AID(CaMep2; 442D), 5AH3 (CaMep2; R452D/S453D) and 5FUF (CaMep2; S453D).

**How to cite this article:** van den Berg, B. *et al.* Structural basis for Mep2 ammonium transceptor activation by phosphorylation. *Nat. Commun.* 7:11337 doi: 10.1038/ncomms11337 (2016).

## Supplementary Material

Supplementary Information Supplementary Figures 1-6 and Supplementary Figures 1-2.
