# Supplementary material for: Structural basis for Mep2 ammonium transceptor activation by phosphorylation
Source: Nat Commun. 2016 Apr 18;7:11337. doi: 10.1038/ncomms11337 (PMC4852598; doi:10.1038/ncomms11337)
Supplement: Supplementary Information — Supplementary Figures 1-6 and Supplementary Figures 1-2. [file ncomms11337-s1.pdf]

Supplementary Information

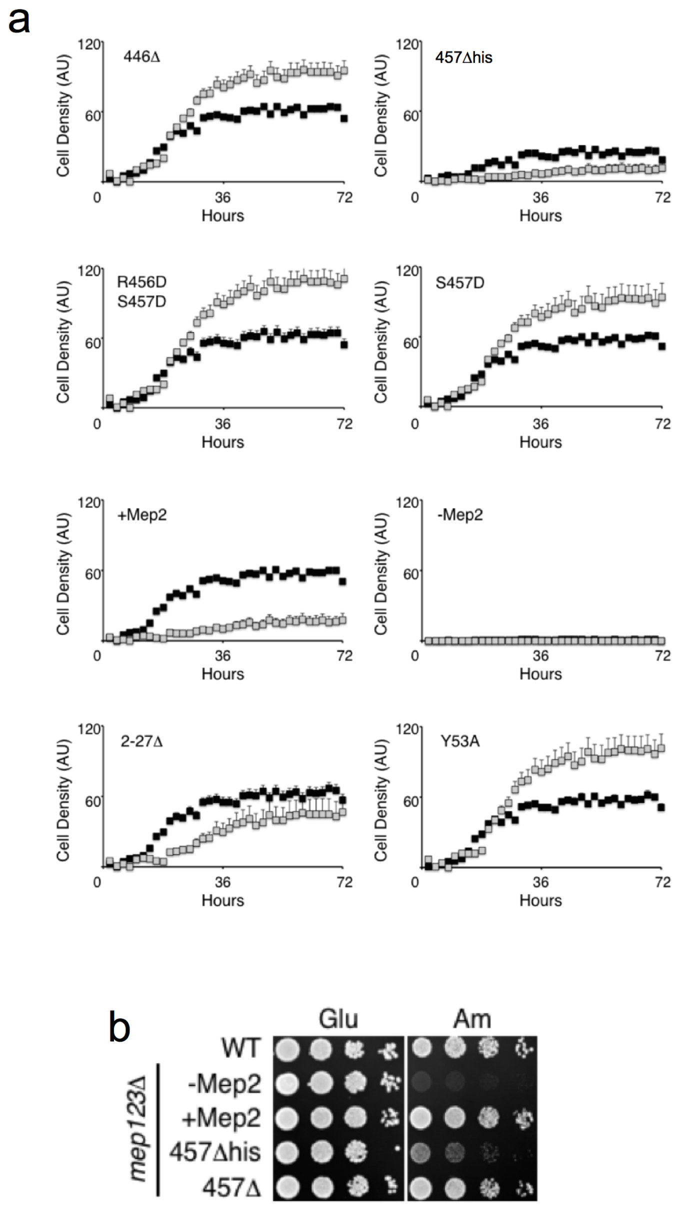

Supplementary Figure 1: Growth of ScMep2 variants on low ammonium medium. The triple *mepΔ* strain (black) and triple *mepΔ npr1Δ* strain (grey) containing plasmids expressing wild-type and variant ScMep2 were grown on medium containing 1mM ammonium sulphate for 72 hours at 30°C. Growth was followed using time course photography. Error bars are the standard deviations for three replicates of each strain. (**b**) The wild type strain (containing a control plasmid) and the triple *mepΔ* strain (containing plasmids expressing wild-type and variant ScMep2) were serially diluted and spotted onto minimal agar plates containing glutamate (Glu; 0.1%) or ammonium sulphate (Am; 1 mM) and grown for 3 days at 30°C.

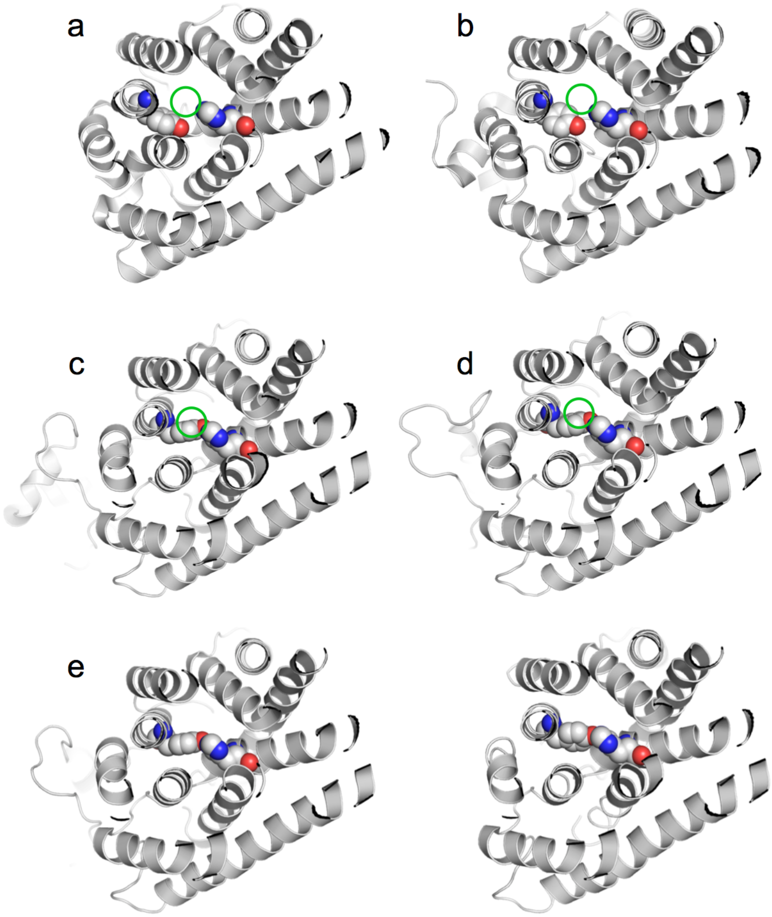

Supplementary Figure 2: Hydrogen bond interactions in Mep2 transporters between the tyrosine residue in TM1 and His2 of the twin-His motif. Shown are cartoon models viewed from the extracellular side with the two residues of interest shown as space-filling models. (**a**) *E. coli* AmtB (PDB ID 1U7G), (**b**) *A. fulgidus* Amt1 (PDB ID 2B2H), (**c**) *C. albicans* Mep2, (**d**) *S. cerevisiae* Mep2, (**e**) *C. albicans* Mep2 DD mutant, (**f**) *C. albicans* Mep2 442Δ mutant. In panels (**a-d**) the channel is indicated with a green circle, showing the blockage in the fungal proteins.

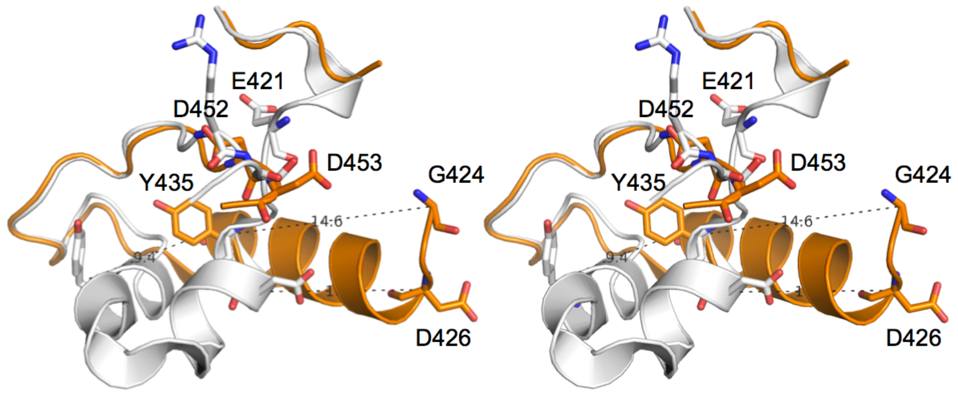

Supplementary Figure 3: Conformational changes in the CTR of the DD mutant. Stereoview superpositions of wild type CaMep2 (white) and the DD mutant (orange). Mutated and conserved CTR residues including those of the ExxGxD motif are shown as stick models and labeled for the DD mutant with the exception of E421, which is disordered and not visible in the structure. Corresponding residues in the two proteins are connected by dashed lines with distances indicated.

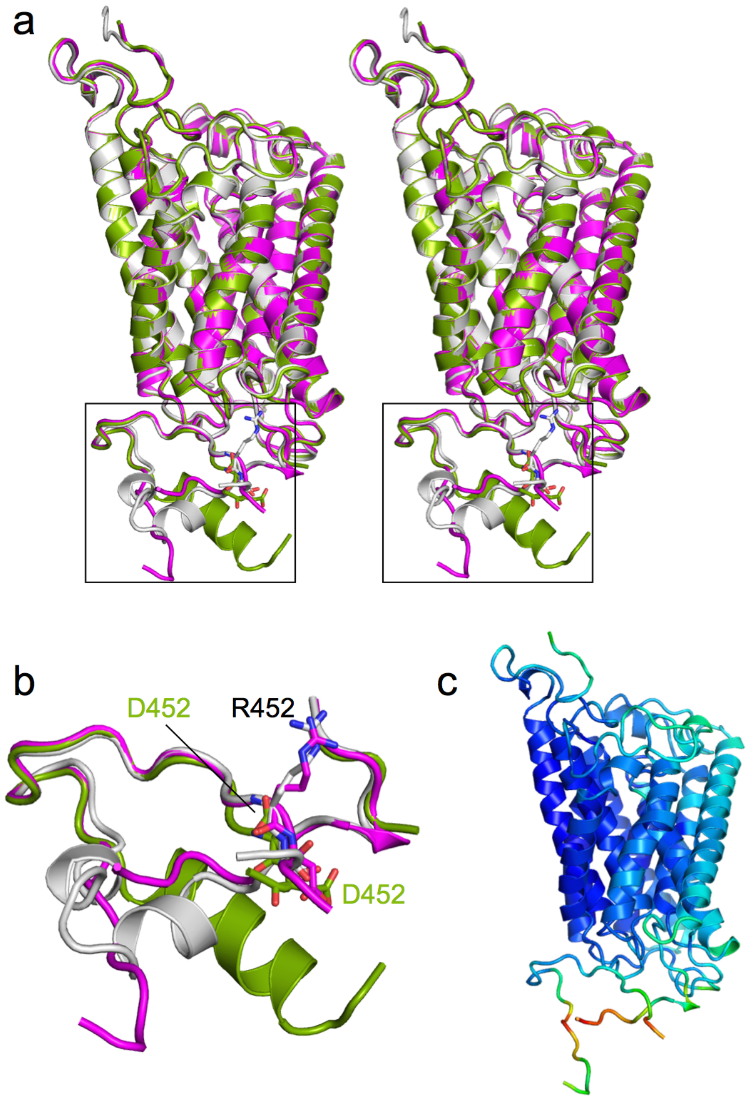

Supplementary Figure 4: X-ray crystal structure of CaMep2 S453D. (**a**) Stereo superposition of CaMep2 WT (grey), CaMep2 DD mutant (green) and CaMep2 S453D ("single D") mutant (magenta). The boxed region indicates the CTR. (**b**) Close-up of the CTRs with residues at positions 452 and 453 shown as stick models. (**c**) Side view of S453D colored by B-factor, showing the lack of order within the CTR.

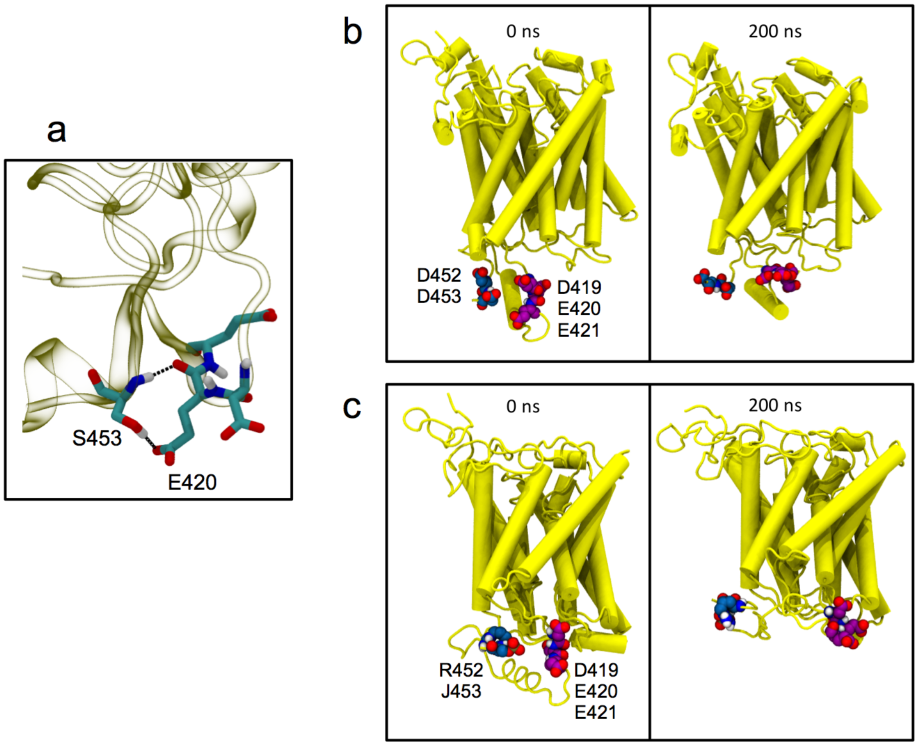

Supplementary Figure 5: MD simulations of WT CaMep2, DD mutant and S453J. (**a**) CTR of WT Mep2 structure after 200 ns simulation. Stable hydrogen bond interactions between Ser453 and Glu420 are indicated by dashed lines. (**b,c**) Side view panels of (**b**) CaMep2 DD and (**c**) S453J after 0 ns (left panels) and 200 ns (right panels) of MD simulation. The acidic pocket residues at positions 419, 420 and 421, as well as residues at positions 452 and 453 are shown as spheres.

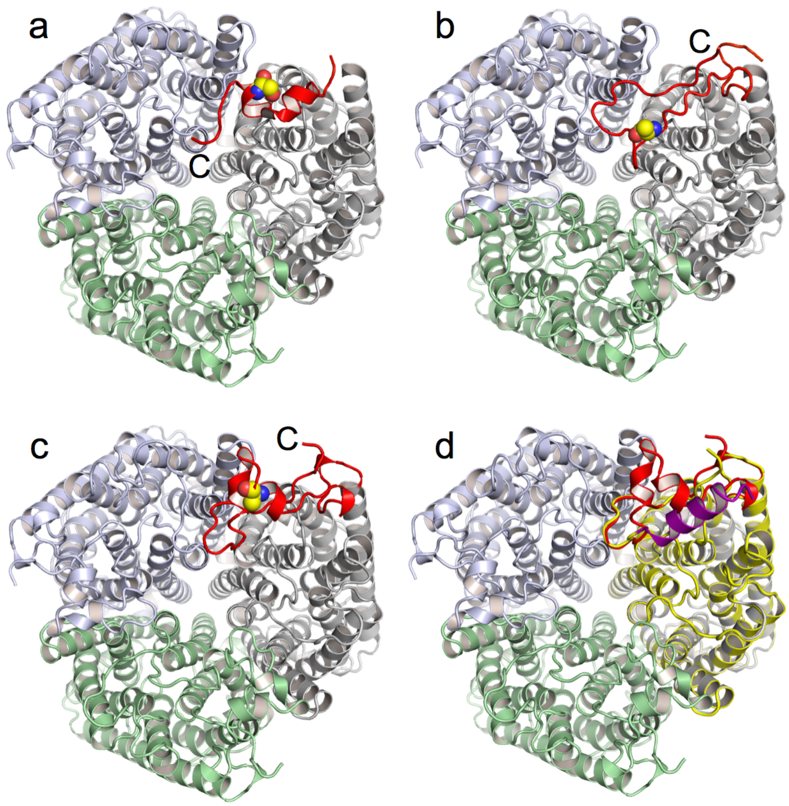

Supplementary Figure 6: Variations within the CTR of ammonium transporters. Trimer views from the cytoplasm with the CTRs of the grey subunits colored red: (**a**) AfAmt-1, (**b**) ScMep2, (**c**) CaMep2 and (**d**) CaMep2 with the DD mutant superposed in yellow. The helix around the ExxGxD domain of the DD mutant is colored purple. In panels (**a-c**), the residue corresponding to T460 of *A. thaliana* Amt1;1 is shown as spheres (carbons; yellow). The C-termini are indicated by "C".

Supplementary Table 1: Data collection and refinement statistics for wild type Mep2 proteins

	ScMep2	CaMep2 #1	CaMep2 #2
**Data Collection**			
DLS Beamline	i02	i02	i24
Wavelength (Å)	0.9796	0.9796	0.969
Space group	$P2_12_12_1$	R3	P3
Cell dimensions (a,b,c)	103.7,232.4,279.2	104.9,104.9,160.7	100.2,100.2,110.0
(α,β,γ)	90,90,90	90,90,120	90,90,120
Molecules/AU	9	1	2
Solvent content (%)	64	61	59
Resolution (Å)	49-3.2	44-1.47	30-1.64
$I/\sigma I$	9.6/2.0	14.9 (2.5)	20.0 (2.0)
Completeness	99.9 (100)	99.8 (99.5)	99.7 (97.2)
Redundancy	4.2 (4.3)	5.6 (5.2)	5.6 (4.8)
Rmerge (%)	9.2 (92)	5.1 (49)	3.9 (62)
Rpim (%)	7.4 (71)	2.6 (27)	2.8 (47)
CC (1/2)	1.00 (0.68)	1.00 (0.84)	1.00 (0.79)
Refinement			
Resolution (Å)	49-3.2	44-1.47	29.6-1.64
Reflections (n)	111734	111840	151123
R_{work}/R_{free} (%)	18.9 (24.1)	13.7 (15.0)	13.6/15.9
Atoms (n)			
protein/solvent/	30531/-	3612/274	3466/536
ligand/detergent	45/-	-/80	-/-
B factors ($Å_2$)			
protein/solvent/	84/-	24/39	32/44
ligand/detergent	135/-	-/77	-/-
R.m.s.d.			
bond lengths (Å)	0.012	0.007	0.007
bond angles (°)	1.15	1.08	1.009
Ramachandran plot (%)			
most favored/disallowed	93.0/0.9	98.7/0.2	98.7 (0.0)
Molprobity clashscore	7.8	2.1	2.3

Supplementary Table 2: Data collection and refinement statistics for *Candida albicans* Mep2 mutants

	S453D	R452D/S453D ("DD")	Δ442
**Data Collection**			
DLS Beamline	i03	i04-1	i04-1
Wavelength (Å)	0.979	0.92	0.92
Space group	$R3_2$	$P6_322$	$R3_2$
Cell dimensions (a,b,c)	109.2,109.2,340.4	112.7,112.7,251.1	114.7,114.7, 290.0
(α,β,γ)	90,90,120	90,90,120	90,90,120
Molecules/AU	1	1	1
Solvent content (%)	67	71	67
Resolution (Å)	30-2.07	49-2.4	97-3.4
$I/\sigma I$	17.1 (2.0)	7.6 (1.9)	14.6 (2.5)
Completeness	99.3 (91.5)	100 (100)	100 (100)
Redundancy	10.9 (10.2)	10.8 (9.9)	9.3 (9.7)
Rmerge (%)	7.4 (70)	18 (99)	9.7 (117)
Rpim (%)	3.4 (33)	8.1 (48)	4.9 (59)
CC (1/2)	1.00 (0.89)	1.00 (0.84)	0.99 (0.82)
Refinement			
Resolution (Å)	30-2.07	49-2.4	96-3.4
Reflections (n)	48392	37561	10439
R_{work}/R_{free} (%)	16.2 (17.4)	17.4 (20.0)	23.6 (28.9)
Atoms (n)			
protein/solvent/	3459/122	3427/129	3041/-
ligand/detergent	-/94	-/91	-/-
B factors ($Å^2$)			
protein/solvent/	49/53	40/44	132/-
ligand/detergent	-/90	-/86	-/-
R.m.s.d.			
bond lengths (Å)	0.008	0.008	0.009
bond angles (°)	0.85	1.06	1.28
Ramachandran plot (%)			
most favored/disallowed	98.0/0.3	97.7/0.0	84.6/3.0
Molprobity clashscore	2.3	1.9	13.9
